# Biological and Molecular Characterization of Two Closely Related Arepaviruses and Their Antagonistic Interaction in *Nicotiana benthamiana*

**DOI:** 10.3389/fmicb.2021.755156

**Published:** 2021-10-18

**Authors:** Yaodi Wang, Wentao Shen, Zhaoji Dai, Bei Gou, Hongjun Liu, Weiyao Hu, Li Qin, Zengping Li, Decai Tuo, Hongguang Cui

**Affiliations:** ^1^Key Laboratory of Green Prevention and Control of Tropical Plant Diseases and Pests, Ministry of Education and College of Plant Protection, Hainan University, Haikou, China; ^2^Institute of Tropical Bioscience and Biotechnology, Chinese Academy of Tropical Agricultural Sciences, Haikou, China

**Keywords:** infectious cDNA clone, pathological properties, viral accumulation, functional compatibility, virus–virus interactions, *Potyviridae*

## Abstract

Previously, our group characterized two closely related viruses from *Areca catechu*, areca palm necrotic ringspot virus (ANRSV) and areca palm necrotic spindle-spot virus (ANSSV). These two viruses share a distinct genomic organization of leader proteases and represent the only two species of the newly established genus *Arepavirus* of the family *Potyviridae*. The biological features of the two viruses are largely unknown. In this study, we investigated the pathological properties, functional compatibility of viral elements, and interspecies interactions in the model plant, *Nicotiana benthamiana*. Using a newly obtained infectious clone of ANRSV, we showed that this virus induces more severe symptoms compared with ANSSV and that this is related to a rapid virus multiplication *in planta*. A series of hybrid viruses were constructed *via* the substitution of multiple elements in the ANRSV infectious clone with the counterparts of ANSSV. The replacement of either 5′-UTR-HCPro1–HCPro2 or CI effectively supported replication and systemic infection of ANRSV, whereas individual substitution of P3-7K, 9K-NIa, and NIb-CP-3′-UTR abolished viral infectivity. Finally, we demonstrated that ANRSV confers effective exclusion of ANSSV both in coinfection and super-infection assays. These results advance our understanding of fundamental aspects of these two distinct but closely related arepaviruses.

## Introduction

*Arepavirus* is a newly established genus under the family *Potyviridae* and consists of two virus species, namely, *areca palm necrotic spindle-spot virus* (ANSSV) and *areca palm necrotic ringspot virus* (ANRSV) (Wylie et al., 2017; Yang et al., 2018, 2019; Cui and Wang, 2019). The two viruses exhibit a distinctive organization in the genomic 5′-terminus that encodes a unique pattern of leader proteases, that is, two cysteine proteases in tandem (HCPro1–HCPro2) (Qin et al., 2021). It has been highlighted that both viruses share 76.2 and 88.6% amino acid (aa) identities at the large polyprotein and coat protein (CP) levels, respectively (Yang et al., 2019). In addition, the ANRSV is highly prevalent with an average incidence rate of 19% in the field and is associated with severe necrotic ringspot symptoms. In contrast, only one ANSSV isolate (to date) has been documented (Yang et al., 2018). Nevertheless, the fundamental aspects of the two closely related arepaviruses, such as pathological properties, functional compatibility of viral elements, and interspecies interactions, are largely unknown.

Swapping viral elements between closely related viruses (or different strains of the same virus), with subsequent determination of the biological alterations, is a common approach to examine functional roles of viral factors in viral infection. This strategy has been widely used to investigate pathogenesis-related biological phenotypes for viruses in the *Potyviridae* family (referred to as potyvirids) (Redondo et al., 2001; Salvador et al., 2008; Decroocq et al., 2009; Wen et al., 2011; Maliogka et al., 2012b; Carbonell et al., 2013; Tian and Valkonen, 2013; Tatineni et al., 2017). For instance, using two plum pox virus (PPV)-C isolates displaying different host-adaption features, viral factors involved in host adaption were determined, and single aa changes in the 6K1-CI region were demonstrated to have trade-off effects on pathogenesis in alternative hosts (Calvo et al., 2014). The VPg of tobacco etch virus is the determinant in rescuing viral infectivity on *Capsicum* genotype *pvr1*^2^, likely mediated through the *pvr1*^2^ eIF4E/VPg interaction (Perez et al., 2012). Notably, the functional compatibility of viral elements between closely related potyvirids (such as these two arepaviruses) has been scarcely studied.

Virus–virus interactions in mixed infections have long been paid considerable attention, and research has progressed substantially in the past several decades. In general, virus interactions, which are categorized as synergistic or antagonistic, are expressed by two (or more) unrelated or closely related viruses (Syller, 2012; Mascia and Gallitelli, 2016; Syller and Grupa, 2016; Moreno and López-Moya, 2020). Usually, potyvirids interact synergistically with distantly related viruses in other families, leading to economically damaging diseases. A classic example is the potato virus X/potyviral synergistic disease (Pruss et al., 1997). Notably, the mixed infection of two or more potyvirids (belonging to either the same or different genus) in a synergistic manner also occurs. For instance, two distinct but closely related viruses in the genus *Ipomovirus*, cassava brown streak virus and Ugandan cassava brown streak virus, synergistically cause cassava brown streak disease, a risk to food security and a major threat to the cassava value chain (Rey and Vanderschuren, 2017; Jacobson et al., 2018). The wheat streak mosaic virus (*Tritimovirus*) and Triticum mosaic virus (*Poacevirus*) cause synergistic mixed infection in cultivar-specific wheat. Antagonistic interactions universally exist between different strains of the same virus or closely related viruses; these interactions have been utilized to develop powerful management strategies against plant-infecting viral pathogens (referred to as mild strain cross-protection) (Ziebell and Carr, 2010; Ziebell and MacDiarmid, 2017; Pechinger et al., 2019). Therefore, studying the interactions between two closely related arepaviruses may facilitate our understanding of disease epidemiology and damage and help develop efficient control strategies.

Given that the arepavirus–areca palm pathosystem has not yet been established, in this study, we investigated the pathological properties, functional compatibility of viral elements, and interspecies interactions in the model host—*Nicotiana benthamiana*. First, we developed the infectious complementary DNA (cDNA) clone of an ANRSV and tested its pathogenicity in comparison with the closely related ANSSV. Furthermore, the functional compatibility of viral elements was assessed by individually replacing different genomic segments in ANRSV with their counterparts from ANSSV. Finally, co-infection and super-infection assays were used to examine virus interactions *in planta*.

## Materials and Methods

### Virus Isolate and Plant Materials

Fresh leaf tissues from one ANRSV-infected areca palm tree at the 5-year-old stage in a commercial growing orchard in Dingan, Hainan, were sampled for cloning full-length genome of ANRSV and subsequent generation of infectious cDNA clone. This virus isolate was referred to as ANRSV-ZYZ. The experimental host *N*. *benthamiana* was maintained in a growth cabinet set to 16-h light/25∘C and 8-h darkness/23∘C.

### Determination of Complete Genome Sequence of ANRSV-ZYZ

The determination of the complete genome sequence of ANRSV-ZYZ was essentially the same as that of previously reported ANRSV-XC1 (Yang et al., 2019). In brief, nine overlapping fragments covering nearly the whole genome were cloned and sequenced. The 5′- or 3′-terminal cDNAs of ANRSV-ZYZ were obtained using 5′ or 3′ RACE kits (Invitrogen). The cloning and sequencing of these resulting polymerase chain reaction (PCR) products were essential, as described previously (Yang et al., 2019).

### Construction of Areca Palm Necrotic Ringspot Virus-Derived Complementary DNA Clones

A low-copy mini-binary T-DNA vector (Xiang et al., 1999) was used as the backbone to construct the full-length cDNA clone of ANRSV-ZYZ. In brief, a modified pCB301 backbone with our desired multiple cloning sites (*Stu*I-*Mlu*I-*Bam*HI-*Eco*RI-*Sal*I) was amplified from the plasmid pVPH-green fluorescence protein (GFP)//mCherry (Cui and Wang, 2016) using primer set RSV-V-F/RSV-V-R (Supplementary Table 1). Four pairs of primers (1-F/2-R, 3-F/3-R, 4-F/4-R, and 5-F/5-R) (Supplementary Table 1) were designed to amplify corresponding portions in the ANRSV genome (from the 5′-terminus: S1, S2, S3, and S4), which were separated with *Mlu*I, *Bam*HI, and *Eco*RI sites. The *Eco*RI site was artificially created by mutating the G at nucleotide (nt) position 2,566 into A during primers synthesis, but the mutation did not alter the encoded aa. The fragments S2, S3, S4, and S1 were integrated, step-by-step, into the modified pCB301 vector to generate the full-length cDNA clone of ANRSV-ZYZ (named pRS).

To develop a GFP-tagged ANRSV clone (pRS-G), the GFP-coding region was amplified from pVPH-GFP/mCherry (Cui and Wang, 2016) and engineered into the NIb/CP intercistronic junction *via* overlapping PCR method with corresponding primers listed in Supplementary Table 1. For the release of free GFP during viral infection, the original cleavage sequence “ASKEFQ/MD” at NIb/CP junction (proteolytically processed by NIa-Pro) was, respectively, engineered into NIb/GFP and GFP/CP junctions. In case that the foreign GFP is removed *via* a potential recombination event during viral replication, the corresponding nt sequences of the two peptides differ in seven nts. Similarly, a mCherry-tagged ANRSV clone (pRS-mCh) was created.

Recently, we developed an infectious cDNA clone of ANSSV (named pSS-I), which was tested to be highly infectious in *N. benthamiana* (Qin et al., 2021). To examine the functional compatibility of viral elements between ANRSV and ANSSV, we constructed five hybrid clones *via* substitution of different elements of ANRSV with the counterparts from ANSSV, i.e., pRS-G-N^SS^, pRS-G-M1^SS^, pRS-G-M2^SS^, pRS-G-M3^SS^, and pRS-G-C^SS^. The pRS-G was used as the backbone to construct these clones using overlapping PCR and other standard DNA manipulation technologies. Here, the detailed description for the creation of pRS-G-N^SS^, in which the 5′-UTR-HCPro1–HCPro2 of ANRSV was replaced by the corresponding one of ANSSV, was exemplified (Supplementary Figure 1). Two PCR reactions with pSS-I-G and pRS-G as templates, respectively, were performed by primer sets PCB301-F/N-1R and N-1F/2-R (Supplementary Table 1). A mixture of the resulting PCR products was used as a template for overlapping PCR with primer set PCB301-F/2-R (Supplementary Table 1). The obtained fragment was inserted back into the pRS-G *via Pme*I/*Mlu*I sites. All these constructs were verified by Sanger sequencing.

### Reverse Transcription-Polymerase Chain Reaction and Real-Time Quantitative Reverse Transcription-Polymerase Chain Reaction

For reverse transcription-polymerase chain reaction (RT-PCR) and real-time quantitative RT-PCR assays, total RNAs were extracted from newly expanded leaves using TRNzol Universal Reagents (TIANGEN) and treated with DNase I (Thermo Fisher Scientific). The first-strand cDNAs were synthesized by reverse-transcription reactions using a RevertAid First Strand cDNA Synthesis Kit (Thermo Fisher Scientific) with random hexamer primer. The PCR reactions with primer sets RS8900F/RS9300R and SS9000F/SS9300R (Supplementary Table 1), targeting CP regions of ANRSV and ANSSV, respectively, were performed to monitor viral infection. Real-time quantitative PCR (qPCR) with SuperReal PreMix Plus (SYBR Green) (TIANGEN) was performed and analyzed with the qTOWER^3^ Real-Time PCR Thermol Cycler (Analytic Jena AG) following the manufacturer’s instructions. In pathogenicity test assay, primer sets RS-9200F/RS-9350R (for ANRSV) and SS-8600F/SV-8700R (for ANSSV) (Supplementary Table 1) were designed and used in the real-time qPCR analysis of viral RNA accumulation levels. The actin transcripts by primer set Actin-145F/Actin-145R (Supplementary Table 1) were used as an internal control to normalize the data.

### Agroinfiltration

Fully expanded leaves of *N. benthamiana* seedlings at an indicated stage were subjected to the infiltration of *Agrobacterium tumefaciens* (GV3101) cultures harboring the relevant plasmids. The cultures were adjusted to the desired optical densities at 600 nm (OD_600_) and infiltrated into leaf tissues of *N. benthamiana* plants essentially as described previously (Cui et al., 2013; Cui and Wang, 2017).

### Western Blotting

Total proteins were extracted from fresh leaf tissues of *N. benthamiana* plants (Cui and Wang, 2016), followed by the separation in 12% sodium dodecyl sulfate–polyacrylamide gel electrophoresis and electroblotting onto a polyvinylidene difluoride membrane (Immobilon). An anti-GFP polyclonal antibody (Abcam) was used in immune detection. Goat anti-rabbit immunoglobulin antibody (Abcam) conjugated to horseradish peroxidase was used as the secondary antibody. The hybridization signals in the blotted membranes were detected with the substrates of enhanced chemiluminescence detection reagents (Thermo Fisher Scientific) and visualized under an ImageQuant LAS 4000 mini biomolecular imager (GE Healthcare).

### Ultraviolet Observation and Fluorescence Microscopy

*N. benthamiana* seedlings inoculated with GFP-tagged virus clones were monitored with a LUYOR-3410 Hand-Held UV lamp (LUYOR). All photographs showing fluorescence signals were taken in a dark chamber at the indicated time point. In co-infection assay, an inverted fluorescence microscope (BX53F, OLYMPUS) was used to examine mCherry signals in leaf tissues indicative of the infection with ANRSV-mCh.

### Co-infection Assay

The co-infection assay was performed to examine the interactions between ANRSV and ANSSV *in planta*. The systemically diseased *N. benthamiana* plants, which were previously agroinfiltrated with pSS-I-G or pRS-mCh, were maintained in a growth cabinet as inoculum sources. Both infected leaf tissues with comparable weight and fluorescence areas (mCherry for pRS-mCh; GFP for pSS-I-G) were, respectively, grounded in phosphate buffer in a ratio of 1:10 (g/ml). The homogenates mentioned earlier were mixed in equal volume, followed by immediate rub-inoculation into the third leaves of 30 *N. benthamiana* seedlings at 4- and 5-leaf-stage. As a control, two mixed homogenates, either pSS-I-G or pRS-mCh and virus-free one (from healthy leaf tissue), were, respectively, rub-inoculated into 25 seedlings. For sap inoculation, a single leaf of each plant was rubbed two times from the leaf bottom to the top using an inoculum-dipped forefinger. In co-infection and super-infection assays, two specific primers, Spe-RS-1000R (for ANRSV) and Spe-SS-1900R (for ANSSV), are designed on the basis of nt sequence differences in the HCPro1–HCPro2 region (Supplementary Figure 2). The Spe-RS-1000R and Spe-SS-1900R (for ANSSV), pairing with primers 1-F and SS900F, are used for specific RT-PCR detection of ANRSV and ANSSV.

### Super-Infection Assay

To examine whether the ANRSV interferes with ANSSV *in planta*, the super-infection assay (pre-infection with ANSSV, followed by challenging inoculation with ANRSV) was performed. First, *N. benthamiana* plants were agroinfiltrated with pSS-I-G, and systemically infected leaves were used as inoculum sources. The fresh leaf tissues from diseased (pSS-I-G) and healthy plants were, respectively, grounded in phosphate buffer in a ratio of 1:10 (grams per milliliter). The resulting homogenates were mechanically inoculated into 100 *N. benthamiana* seedlings at 3- to 4-leaf-stage, including 60 for pSS-I-G and 40 for virus-free homogenate. These plants were equally divided into two groups. Ten days later, one group of plants were subjected to challenging rub-inoculation with ANRSV-containing homogenate, and the other group plants as control were inoculated with virus-free homogenate.

## Results

### Full-Length Complementary DNA Clone of ANRSV-ZYZ Is Infectious in *N. benthamiana*

**FIGURE 1:**
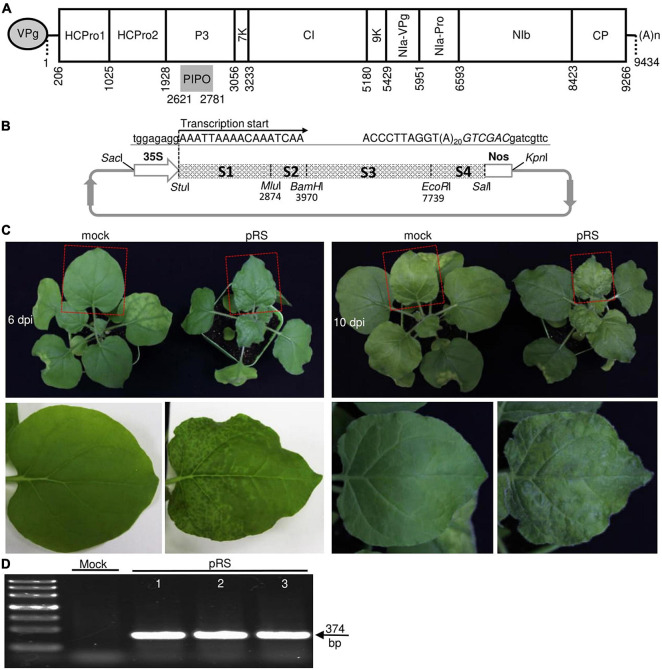
Development of an infectious cDNA clone of ANRSV-ZYZ. **(A)** Schematic diagram showing genomic organization of ANRSV-ZYZ. Oval at 5′-end represents genome-linked viral protein VPg. (A)n at 3′-end indicates poly (A) tail. 5′- and 3′-untranslated regions are denoted by two short horizontal lines. Large rectangle box (nt 206–9,266) and short gray bar (nt 2,621–2,781) indicate corresponding long ORF and small ORF (PIPO) embedded in P3-coding region. **(B)** Schematic representation of full-length cDNA clone of ANRSV-ZYZ (pRS). Four fragments (from 5′-terminus: S1, S2, S3, and S4) covering entire genome of ANRSV-ZYZ were engineered between 35S promoter and Nos terminator in a modified T-DNA vector pCB301-35S-Nos (Cui and Wang, 2016). Transcription start site is shown. nts belonging to pCB301 backbone and virus are indicated in lowercase and uppercase, respectively. *Sal*I site immediately following poly (A) tail is shown in italicized uppercase. **(C)** Infectivity test of pRS in *N. benthamiana*. Representative photographs of agroinfiltrated plants were taken at 6 and 10 dpi. Systemic leaves indicated by red box are closely viewed. Mock, empty vector control. **(D)** RT-PCR detection of ANRSV. Systemic leaf tissues of agroinfiltrated plants were sampled at 10 dpi and subjected to RT-PCR assay with primer set RS8900F/RS9300R.

To study the pathological properties of ANRSV, we developed the full-length cDNA clone of an ANRSV isolate (ANRSV-ZYZ). We first determined the complete genome sequence of ANRSV-ZYZ (GenBank accession no. MZ209276), followed by its annotation with reference to a previously documented isolate ANRSV-XC1 (Yang et al., 2019; Qin et al., 2021). The complete 9,434-nt genome sequence of ANRSV-ZYZ, excluding the 3′-terminal poly (A) tail, contains a single long open reading frame (ORF) and another relatively short ORF (PIPO) that results from RNA polymerase slippage in the P3-coding region (Chung et al., 2008; Olspert et al., 2015; Rodamilans et al., 2015; White, 2015). The two deduced polyproteins contain the predictable cleavage sites by three virus-encoded proteases (P1, HCPro, and NIa-Pro) for the generation of 11 mature proteins (Figure 1A).

The full-length cDNA of ANRSV-ZYZ was engineered into a low-copy mini-binary T-DNA backbone pCB301 (Xiang et al., 1999) and driven by a 35S promoter (Figure 1B). Transcription started from the first nt A of the ANRSV-ZYZ genome (Figure 1B), allowing the precise production of an authentic 5′-end. To mimic the true 3′-end of the viral genome during viral infection, a 20-nt poly (A) sequence was engineered immediately downstream of 3′-UTR (Figure 1B). To test the infectivity of the resulting cDNA clone of ANRSV-ZYZ (pRS), 12 *N. benthamiana* seedlings at the 3- to 5-leaf-stage were infiltrated with a pRS-harbored agrobacterial culture at OD_600_ of 0.5. All plants inoculated with pRS exhibited typical vein clearing symptoms in newly expanded leaves at 6 days post-infiltration (dpi) and severe rugosity and chlorosis at 10 dpi (Figure 1C); these symptoms were absent in mock control plants. The presence of ANRSV in these diseased plants was confirmed by an RT-PCR assay targeting the partial CP cistron of ANRSV (Figure 1D). Thus, the newly created cDNA clone of ANRSV was observed to be highly infectious in the experimental host, *N. benthamiana*.

### Pathological Properties of the Two Closely Related Arepaviruses

**FIGURE 2:**
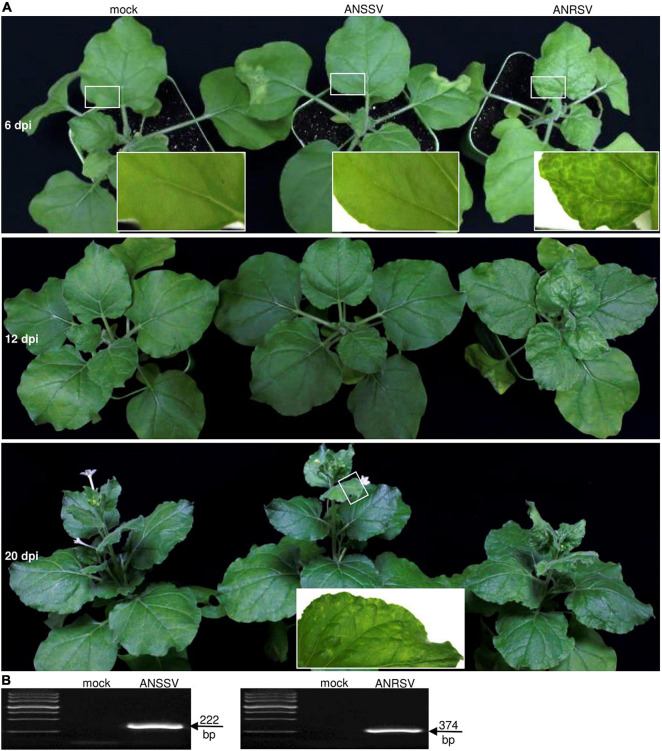
Pathogenicity comparison between two closely related arepaviruses (ANRSV and ANSSV) in *Nicotiana benthamiana*. **(A)** Symptom phenotypes triggered by ANRSV and ANSSV. Representative photographs of agroinfiltrated plants were taken at 6, 12, and 20 dpi. Close views of marked regions in white rectangle are shown. Mock, empty vector control. **(B)** RT-PCR detection of ANSSV and ANRSV. Newly developed leaves of agroinfiltrated plants were sampled at 20 dpi and subjected to RT-PCR assay with primer sets RS8900F/RS9300R (for ANRSV) and SS9000F/SS9300R (for ANSSV).

Another areca palm-infecting virus, ANSSV, the type virus species of the genus *Arepavirus*,^1^ is a close relative of ANRSV. The two viruses have the same genomic organization and share relatively high sequence identities at either the whole genome or *CP* level (Yang et al., 2019). Here, we compared the pathological properties between them. Two agrobacterial cultures (OD_600_, 1.0) containing corresponding cDNA clones of ANSSV (pSS-I) and ANRSV (pRS) were each infiltrated into 12 *N. benthamiana* seedlings at the 5- to 6-leaf-stage. All plants inoculated with pRS exhibited typical vein clearing symptoms in the top new leaves at 6 dpi and severe chlorosis and rugosity at 12 dpi (Figure 2A). At 20 dpi, the plants inoculated with pSS-I started to display mild chlorosis in the top new leaves. In the contrary, the pRS-inoculated plants display dwarfism at 20 dpi (Figure 2A). As expected, RT-PCR confirmed the presence of corresponding viruses in these agroinfiltrated plants at 20 dpi (Figure 2B). Thus, the two arepaviruses displayed distinct symptomatology in *N. benthamiana*.

### Symptom Severity Conveyed by Areca Palm Necrotic Ringspot Virus Is Associated With a Rapid Viral Accumulation *in planta*

**FIGURE 3:**
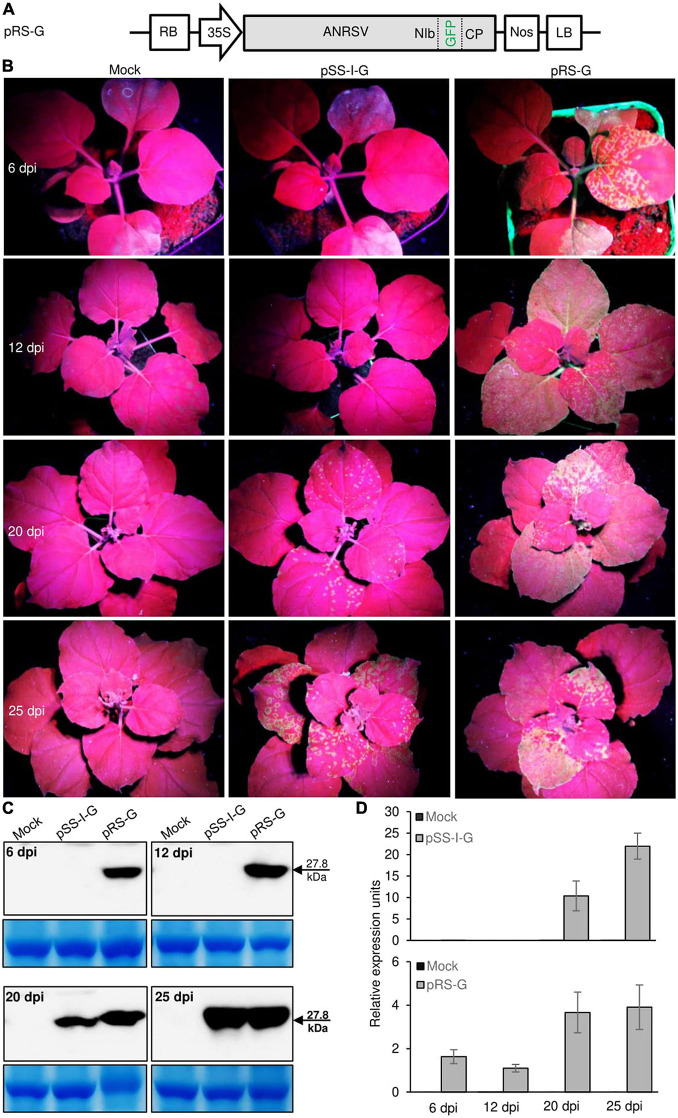
Pathogenicity discrepancy between ANRSV and ANSSV is associated with viral accumulation *in planta*. **(A)** Schematic diagram of GFP-tagged ANRSV clone (pRS-G). For pRS-G, a GFP-coding region was engineered into NIb/CP intercistronic junction. **(B)** Infectivity comparison between GFP-tagged ANRSV (pRS-G) and ANSSV clones (pSS-I-G) in *N. benthamiana* plants. Representative photographs were taken under an ultraviolet lamp at 6, 12, 20, and 25 dpi. Mock, empty vector control. **(C)** Immunoblot analysis of GFP accumulation levels in *N. benthamiana* plants inoculated with pRS-G and pSS-I-G. Systemic leaf tissues in *N. benthamiana* plants were sampled at indicated time points. Immunoblot analysis using anti-GFP polyclonal antibody was performed to determine GFP accumulation levels. Coomassie blue staining of large subunit of rubisco was used as a loading control for immunoblot. **(D)** Real-time qPCR analysis of viral RNA accumulation levels. Systemic leaf tissues from plants inoculated with pRS-G and pSS-I-G were sampled at indicated time points and subjected to real-time qPCR analysis of viral genomic RNA accumulation levels.

To visually monitor ANRSV infection *in planta*, the foreign GFP cistron was engineered into the NIb/CP intercistronic junction to generate the GFP-recombinant ANRSV clone, pRS-G (Figure 3A). To examine whether the discrepancy in pathogenicity between ANRSV and ANSSV is associated with viral accumulation, two agrobacterial cultures (OD_600_, 1.0) harboring corresponding GFP-tagged ANSSV (pSS-I-G) (Qin et al., 2021) and ANRSV clones (pRS-G) were infiltrated into 15 *N. benthamiana* seedlings at the 4- to 5-leaf-stage each. All plants inoculated with pRS-G displayed strong GFP signals along the veins of newly expanded leaves at 6 dpi and systemic signals at 12 dpi (Figure 3B). However, the plants inoculated with pSS-I-G displayed scattered fluorescence spots at 20 dpi and systemic GFP signals at 25 dpi (Figure 3B). This result was reconfirmed by Western blot analysis of GFP accumulation levels (Figure 3C). Consistently, the real-time qPCR analysis revealed that ANRSV has a higher dynamic in genomic RNA accumulation (Figure 3D).

To examine whether the discrepancy in pathogenicity between ANRSV and ANSSV (associated with viral multiplication) is caused by differential *Agrobacterium*-mediated transfection efficiency, homogenates were prepared at 30 dpi from leaves systemically infected with ANRSV-G (pRS-G) and ANSSV-G (pSS-I-G), followed by rub-inoculation into the third leaves of more than 10 *N. benthamiana* plants each at the 5- to 6-leaf-stage. Time-course observation results revealed a similar trend with the *Agrobacterium*-mediated transfection assay, either in symptom development or viral multiplication, as that obtained with the GFP signals (Supplementary Figure 3). Collectively, the pathogenicity discrepancy between the two closely related arepaviruses is associated with the velocity of viral multiplication *in planta*.

### Individual Substitution of Viral Elements in the Areca Palm Necrotic Ringspot Virus Genome With the Counterparts of Areca Palm Necrotic Spindle-Spot Virus Has Differential Effects on Viral Infectivity

**FIGURE 4:**
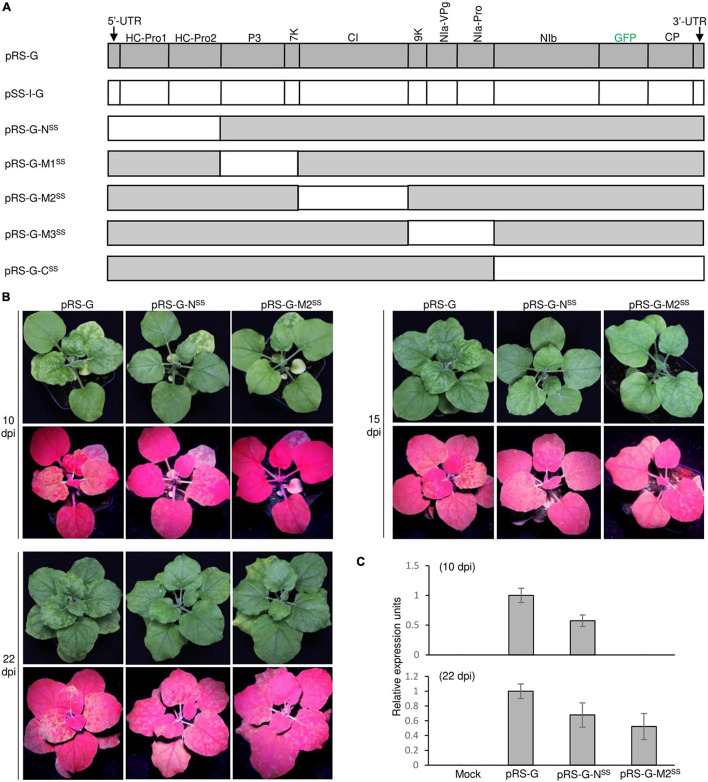
Infectivity test of a series of ANRSV-derived hybrid clones in *N*. *benthamiana*. **(A)** Schematic representation of ANRSV-derived hybrid clones. These clones were generated *via* substitution of different viral elements in ANRSV genome with their counterparts from ANSSV. These elements are (from 5′-terminus): 5′-UTR-HCPro1-HCPro2, P3-7K, CI, 9K-NIa, and NIb-CP-3′-UTR. **(B)** Infectivity test of indicated clones. Representative photographs of agroinfiltrated plants were taken at 10, 15, and 22 dpi. **(C)** Real-time qPCR analysis of viral RNA accumulation levels in *N. benthamiana* plants agroinfiltrated with indicated clones. Newly expanded leaves were sampled at 10 and 22 dpi and subjected to real-time qPCR quantification of viral genomes.

To examine the functional compatibility of viral elements between the two closely related arepaviruses, we constructed a series of hybrid virus clones *via* individual substitution of viral elements in the ANRSV genome with their counterparts from ANSSV. For potyvirids, the first two viral proteins encoded by the genomic N-terminus (upstream P3-coding region) might confer certain biological relevance in a coordinated manner (Pasin et al., 2014; Revers and García, 2015; Cui and Wang, 2019; Yang et al., 2021). Additionally, the incomplete processing for cleavage sites at P3/6K1, 6K2/NIa-Pro, and NIa-VPg/NIa-Pro junctions by NIa-Pro results in precursors such as P3-6K1, NIa, and 6K2-NIa that are implicated in viral infection (Riechmann et al., 1995; Urcuqui-Inchima et al., 2001; Cotton et al., 2009; Cui and Wang, 2016). Following the criterion of maintaining these functional intermediates, five hybrid virus clones (i.e., pRS-G-N^SS^, pRS-G-M1^SS^, pRS-G-M2^SS^, pRS-G-M3^SS^, and pRS-G-C^SS^) were created (Figure 4A). Agrobacterial cultures corresponding to these clones at OD_600_ of 0.5 were each inoculated into at least eight *N. benthamiana* seedlings each at the 3- and 4-leaf-stage.

For the convenience of description, the resulting viruses from these hybrid clones were designated as ANRSV-G-N^SS^, ANRSV-G-M1^SS^, ANRSV-G-M2^SS^, ANRSV-G-M3^SS^, and ANRSV-G-C^SS^, and viruses from wild-type virus clones (pRS-G and pSS-I-G) were referred to as ANRSV-G and ANSSV-G. Similar to ANSSV-G, all these five hybrid viruses did not trigger discernable symptomatic phenotype at 10, 15, and 22 dpi (Figure 4B and Supplementary Figure 4). Furthermore, the GFP signals were monitored at these time points. All plants inoculated with pRS-G-N^SS^ displayed a similar fluorescence pattern as those of the pRS-G plants at 10, 15, and 22 dpi, albeit slightly weaker than the latter (Figure 4B). For pRS-G-M2^SS^ plants, scattered fluorescence spots appeared at 15 dpi and systemic fluorescence signals at 22 dpi, indicating that ANRSV-G-M2^SS^ accumulates at a slower rate than ANRSV-G but faster than ANSSV-G (Figure 4B and Supplementary Figure 4). Consistent results in viral RNA accumulation levels were obtained *via* real-time qPCR analysis of viral genomic RNA at 10 and 22 dpi (Figure 4C). Together, the results corroborate that the 5′-UTR, HCPro1, HCPro2, and CI factors from the heterologous virus, ANSSV, are able to effectively support the multiplication and systemic infection of ANRSV.

Remarkably, all plants inoculated with the other hybrid virus clones (pRS-G-M1^SS^, pRS-G-M3^SS^, and pRS-G-C^SS^) did not show any distinguishable symptoms and fluorescence signals (Supplementary Figure 4). Hence, these virus clones were lethal in *N. benthamiana*, implying that the three elements (i.e., P3-7K, 9K-NIa, and NIb-CP) of ANRSV function in a virus-specific manner.

### Areca Palm Necrotic Ringspot Virus Interferes With the Colonization of Areca Palm Necrotic Spindle-Spot Virus During Viral Co-infection

**FIGURE 5:**
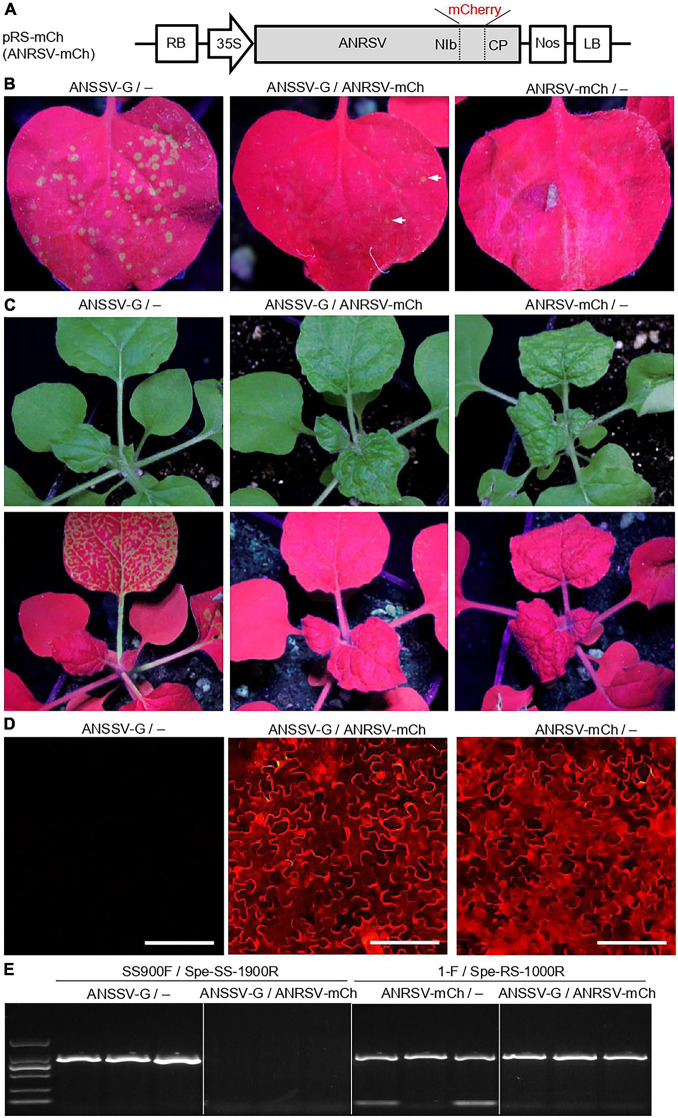
ANRSV interferes with colonization of ANSSV during viral co-infection. **(A)** Schematic diagram of a mCherry-tagged ANRSV clone (pRS-mCh). For pRS-mCh, a mCherry-coding region was engineered into NIb/CP intercistronic junction. **(B)** Green fluorescence observation in rub-inoculated leaves with mixed homogenates of ANSSV-G/−, ANSSV-G/ANRSV-mCh, and ANRSV-mCh/−. Virus-free homogenate from a healthy plant is indicated by symbol “−.” Representative photographs were taken in a dark chamber under an ultraviolet lamp at 3 days post-rub-inoculation (dpri). **(C)** Symptom phenotype and green fluorescence distribution in *N. benthamiana* plants rub-inoculated with indicated mixed homogenates. Representative photographs were taken in daylight (upper) and under an ultraviolet lamp (lower) at 8 dpri. **(D)** Cellular red fluorescence pattern in rub-inoculated leaves with indicated mixed homogenates at 8 dpri. Distribution of mCherry signals was examined using an inverted fluorescence microscope (BX53F, OLYMPUS). Bar, 100 μm. **(E)** Specific RT-PCR detection of ANSSV-G and ANRSV-mCh in *N. benthamiana* plants rub-inoculated with indicated mixed homogenates. Newly developed leaves of rub-inoculated plants were sampled at 8 dpri and subjected to specific RT-PCR detection of ANRSV and ANSSV with primer sets 1-F/Spe-RS-1000R and SS900F/Spe-SS-1900R, respectively.

As ANRSV and ANSSV are two distinct but closely related viruses in the genus *Arepavirus*, we speculated the likely inter-species interaction pattern between them. Thus, we first performed a co-infection assay. For this, we constructed a mCherry-tagged ANRSV clone (pRS-mCh) (Figure 5A), and the corresponding recombinant virus was designated as ANRSV-mCh. Similar to pRS-G, the pRS-mCh was efficiently infectious in *N. benthamiana* (data not shown). The mixed homogenates of ANSSV-G and ANRSV-mCh were rub-inoculated into 30 *N. benthamiana* plants. As the control, the mixed homogenates, either ANSSV-G or ANRSV-mCh and virus-free (indicated by the symbol “−”) ones, were inoculated into 25 plants each.

Four days later, several bright green fluorescence foci were observed in the inoculated leaves of ANSSV-G/− control plants (48.8 foci on average per leaf) (Figure 5B). In contrast, the plants treated with ANRSV-mCh/− exhibited scattered and weak fluorescence foci at this time point (4.4 foci on average). At 8 dpi, all plants treated with ANSSV-G/− showed strong GFP signals in the top leaves under a UV lamp, indicating the systemic infection of ANSSV-G (Figure 5C). No GFP signals were observed in either ANSSV-G/ANRSV-mCh or ANRSV-mCh/− plants; however, these plants exhibited typical ANRSV-induced symptoms such as severe vein clearing and rugosity symptoms in the top leaves. The systemic infection by ANRSV-mCh in these plants was confirmed by fluorescence microscopy (Figure 5D).

To discriminate the presence of ANRSV and ANSSV *in planta*, an assay for specific RT-PCR detection of ANRSV and ANSSV was established (Supplementary Figure 2). The results of RT-PCR further corroborated the presence of ANSSV-G in plants treated with ANSSV-G/− and of only ANRSV-mCh in plants treated with either ANSSV-G/ANRSV-mCh or ANRSV-mCh/− (Figure 5E). Similar results were obtained from two additional independent experiments.

### Secondary Virus, Areca Palm Necrotic Ringspot Virus, Confers Efficient Exclusion of the Primarily Infected Areca Palm Necrotic Spindle-Spot Virus *in planta*

**FIGURE 6:**
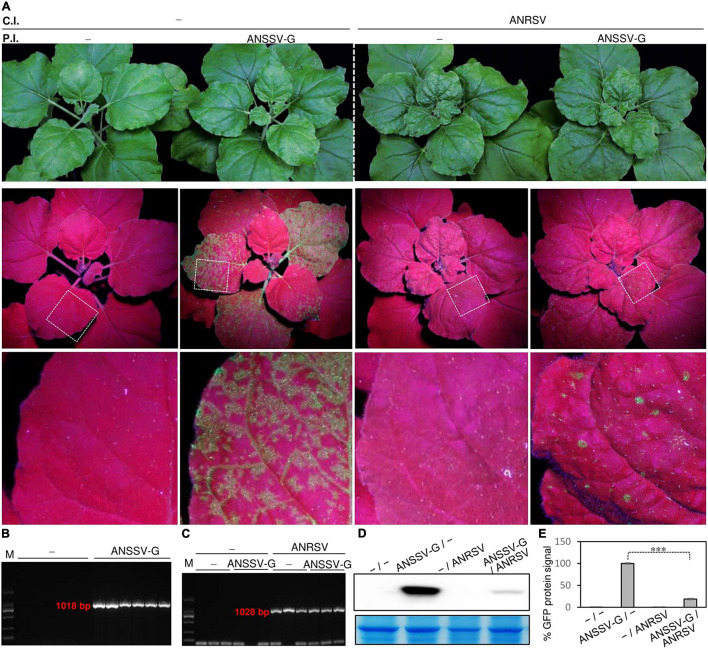
Preexistence of ANSSV fails to defend against challenging ANRSV in *N. benthamiana*. **(A)** Preexistence of ANSSV *in planta* fails to interfere with infection progression of secondary ANRSV. A total of 60 *N. benthamiana* seedlings at 3- to 4-leaf-stage were subjected to primary rub-inoculation with ANSSV-G. As a control, another 60 plants were inoculated with virus-free homogenate. Types discussed earlier of pre-treated plants were equally divided into two groups. After 15 days, one group of plants were subjected to challenge rub-inoculation with ANRSV-containing homogenate and other group with virus-free homogenate. Representative photographs were taken at 15 days post-challenging inoculation (dpci). P.I., primary inoculation; C.I., challenge inoculation; −, homogenate from healthy leaf tissues. **(B)** Specific RT-PCR detection of ANSSV-G in pre-treated *N. benthamiana* plants. Newly expanded leaves from plants primarily inoculated with ANSSV-G or “−” were sampled at 8 dpi and subjected to specific RT-PCR detection of ANSSV-G with primer set SS900F/Spe-SS-1900R. **(C)** Specific RT-PCR detection of ANRSV in challenge-inoculated plants. Newly expanded leaf tissues of plants challenge inoculated with ANRSV and “−” were sampled at 8 dpci and subjected to specific RT-PCR detection of ANRSV with primer set 1-F/Spe-RS-1000R. **(D)** Immunoblot analysis of GFP accumulation in *N. benthamiana* plants treated with ANSSV-G/− and ANSSV-G/ANRSV. Systemic leaf tissues in *N. benthamiana* plants were sampled at 15 dpci. Immunoblot analysis using anti-GFP polyclonal antibody was performed to determine GFP accumulation levels. Coomassie blue staining of large subunit of rubisco was used as loading control. **(E)** Quantitative analysis of GFP signals shown in panel **(D)**. Signal intensity values are presented as mean ± SD (*n* = 3). Average value for ANSSV-G/− was designated as 100% to normalize data. Statistically significant differences, determined by an unpaired two-tailed Student’s *t*-test, are indicated by asterisks. ^∗∗∗^*P*< 0.001.

To further examine the interactions between ANRSV and ANSSV *in planta*, a super-infection assay was conducted. A total of 60 *N. benthamiana* seedlings at 3- to 4-leaf-stage were primarily rub-inoculated with the ANSSV-G homogenate (Figure 6A), and another 40 plants were rub-inoculated with virus-free homogenate (indicated by the symbol “−”). After 15 days, all plants inoculated with ANSSV-G exhibited systemic fluorescence distribution (similar to that shown in Supplementary Figure 3), and the presence of ANSSV-G was confirmed by RT-PCR assay (Figure 6B). Immediately, 30 plants pre-infected by ANSSV-G, together with 20 plants pre-inoculated with “−,” were subjected to a challenge inoculation with the ANRSV-containing homogenate. The third leaf of each plant (ANSSV-G), usually displaying extensive fluorescence signals, was rubbed.

At 8 days post-challenge inoculation (dpci), the typical ANRSV-induced symptoms such as vein clearing and rugosity in top leaves were observed in all plants treated with −/ANRSV. A similar symptom phenotype was observed in 26 of 30 plants treated with ANSSV-G/ANRSV (data not shown). The association of these symptoms with ANRSV infection was confirmed by specific RT-PCR detection of ANRSV (Figure 6C). At 15 dpci, all plants treated with either −/ANRSV or ANSSV-G/ANRSV showed typical ANRSV-triggered symptoms (such as severe rugosity) (Figure 6A), indicating the preexisting ANSSV-G did not, at least obviously, interfere with the infection progression of ANRSV.

In contrast to ANSSV-G/− plants, the GFP signal in ANSSV-G/ANRSV plants gradually faded after 8 dpci. Only scattered fluorescence spots, indicative of the infection by ANSSV-G, were observed in the top leaves of ANSSV-G/ANRSV plants at 15 dpci, indicating the primarily infected ANSSV-G was greatly restricted (Figure 6A). In comparison with ANSSV-G/− plants, the substantial reduction in GFP accumulation in ANSSV-G/ANRSV plants at 15 dpci was reconfirmed by Western blot analysis (Figures 6D,E). Conclusively, the ANRSV, albeit being subsequently inoculated, still conferred efficient exclusion of the preexisting ANSSV. The observations were reconfirmed through two additional independent experiments.

## Discussion

Previously, our group identified two closely related arepaviruses (ANRSV and ANSSV) from areca palm trees, with the former being the prevalent virus in the field (Yang et al., 2018, 2019). Additionally, both shared relatively conserved genomic sequences; particularly, the CP identity was slightly above the cut-off value for species demarcation in the family *Potyviridae* (Adams et al., 2005; Wylie et al., 2017). Hence, the two arepaviruses are believed to be closely linked in evolutionary lineage. However, the biological features of the two distinct but closely related viruses are largely unknown. In this study, we performed the related investigations in the model host, *N. benthamiana*, and determined the following: (i) a series of ANRSV-derived cDNA clones were successfully generated, which could serve as valuable tools to study fundamental aspects of arepaviruses; (ii) the viral factors (5′-UTR, HCPro1, HCPro2, and CI) from a heterologous virus (ANSSV) are compatible for replication and systemic infection of ANRSV but not P3-7K, 9K-NIa, and NIb-CP-3′-UTR; and (iii) the ANRSV effectively excludes ANSSV both in co-infection and super-infection assays, indicating that the interactions between them *in planta* are antagonistic. These findings greatly advance our understanding of fundamental aspects of the two distinct but closely related viruses in the new genus *Arepavirus* of the family *Potyviridae*.

Examining the pathological properties of the two closely related arepaviruses is of great interest and will help to develop novel antiviral strategies. In the field, the ANRSV is highly epidemic in nature with an average incidence rate of 19% and is associated with severe necrotic ringspot symptoms (Yang et al., 2019). In contrast, only one ANSSV isolate (to date) has been documented (Yang et al., 2018). The ANSSV-infected areca palm tree exhibits necrotic spindle-spot symptoms at its infancy stage (approximately 2 years old) (Yang et al., 2018). Intriguingly, the typical virus-induced symptoms have not been discriminated in subsequently developed leaves (data not shown). Therefore, the pathological properties of arepaviruses seem complicated in the field. This study characterized the discrepancy in pathogenicity between the two closely related viruses, which is associated with the velocity of viral accumulation *in planta*. However, further testing in natural hosts is required, for which the following aspects should be considered critically: (i) the demarcation criteria for areca palm cultivars have not been established; (ii) the perennial areca palm is unyielding to genetic transformation, limiting the development of transfection methods of arepavirus; and (iii) although only one ANSSV isolate has been documented thus far (Yang et al., 2018), the population of the virus in the field still needs to be investigated.

Investigating the functional compatibility of viral elements between closely related viruses (or different strains of the same virus) facilitates the exploration of the functional roles of viral factors in viral infection. For potyviruses, the primary task executed by leader proteases (P1-HCPro) during viral compatible infection is defending the RNA silencing-mediated immune response. For example, the replacement of HCPro (or P1-HCPro) by other heterologous viral suppressors of RNA silencing in PPV generates viable viruses with different efficiencies in systemic infection (Carbonell et al., 2012; Maliogka et al., 2012a; Rodamilans et al., 2018). The VSRs may be interchangeable, explaining the infectivity of the chimera ANRSV-G-N^SS^. The fact that three of the five chimeras (i.e., ANRSV-G-M1^SS^, ANRSV-G-M3^SS^, and ANRSV-G-C^SS^) were non-infectious is likely because of the complex interactions between the notorious multifunctional potyvirid proteins, especially those involved in virus replication (Sorel et al., 2014; Revers and García, 2015; Cui et al., 2017; Gallo et al., 2018; Shen et al., 2020).

The virus–virus interactions, including synergism and antagonism, are implicated in disease epidemiology, field loss, and development of controlling strategies (Gómez et al., 2009, 2010; Syller, 2012; Mascia and Gallitelli, 2016; Syller and Grupa, 2016; Moreno and López-Moya, 2020). The antagonistic interaction between ANRSV and ANSSV reveals no mixed infection found in the field (Yang et al., 2019). Furthermore, that ANRSV outcompetes ANSSV either in co-infection or super-infection assay likely explains the high prevalence of ANRSV (Yang et al., 2019), although this hypothesis needs to be further tested in the natural host. Another possible explanation is the poor fitness of ANSSV in the model host, leading to its failure in defending against ANRSV, as in the case of the *Citrus tristeza* virus (Harper et al., 2017). Thus, whether the ANSSV could serve as a natural vaccine for cross-protective application mostly depends on its virulence in the natural host.

## Data Availability Statement

The datasets presented in this study can be found in online repositories. The names of the repository/repositories and accession number(s) can be found in the article/Supplementary Material.

## Author Contributions

HC, YW, WS, and ZD conserved and designed the project. YW, WS, and ZD carried out experiments. HC supervised the work. HC, YW, and ZD wrote the manuscript. All authors analyzed, discussed the data, reviewed, and approved the manuscript.

## Conflict of Interest

The authors declare that the research was conducted in the absence of any commercial or financial relationships that could be construed as a potential conflict of interest.

## Publisher’s Note

All claims expressed in this article are solely those of the authors and do not necessarily represent those of their affiliated organizations, or those of the publisher, the editors and the reviewers. Any product that may be evaluated in this article, or claim that may be made by its manufacturer, is not guaranteed or endorsed by the publisher.

## Footnotes

1
https://talk.ictvonline.org/taxonomy/

## Supplementary Material

Supplementary Figure 1 The construction strategy of five ANRSV-derived virus clones.

Supplementary Figure 2 Development of virus-specific primers for RT-PCR detection of ANSSV ANRSV.

Supplementary Figure 3 Time-course observation on viral infection progression in *N. benthamiana* plants rub-inoculated with ANRSV-G and ANSSV-G.

Supplementary Figure 4 Infectivity test of ANRSV-derived clones in *N. benthamiana*.

Supplementary Table 1 Primers list in this study.

## References

[B1] Adams M. J. Antoniw J. F. Fauquet C. M. (2005). Molecular criteria for genus and species discrimination within the family Potyviridae. *Arch. Virol.* 150 459–479. 10.1007/s00705-004-0440-6 15592889

[B2] Calvo M. Malinowski T. García J. A. (2014). Single amino acid changes in the 6K1-CI region can promote the alternative adaptation of Prunus- and Nicotiana-propagated plum pox virus C isolates to either host. *Mol. Plant Microbe Interact.* 27 136–149. 10.1094/MPMI-08-13-0242-R 24200075

[B3] Carbonell A. Dujovny G. García J. A. Valli A. (2012). The cucumber vein yellowing virus silencing suppressor P1b can functionally replace HCPro in plum pox virus infection in a host-specific manner. *Mol. Plant Microbe Interact.* 25 151–164. 10.1094/MPMI-08-11-0216 21970691

[B4] Carbonell A. Maliogka V. I. Pérez J. D. J. Salvador B. León D. S. García J. A. (2013). Diverse amino acid changes at specific positions in the N-terminal region of the coat protein allow plum pox virus to adapt to new hosts. *Mol. Plant Microbe Interact.* 26 1211–1224. 10.1094/MPMI-04-13-0093-R 23745677

[B5] Chung B. Y. W. Miller W. A. Atkins J. F. Firth A. E. (2008). An overlapping essential gene in the Potyviridae. *Proc. Natl. Acad. Sci. U. S. A.* 105 5897–5902. 10.1073/pnas.0800468105 18408156 PMC2311343

[B6] Cotton S. Grangeon R. Thivierge K. Mathieu I. Ide C. Wei T. (2009). Turnip mosaic virus RNA replication complex vesicles are mobile, align with microfilaments, and are each derived from a single viral genome. *J. Virol.* 83 10460–10471. 10.1128/JVI.00819-09 19656892 PMC2753101

[B7] Cui H. Hong N. Wang G. Wang A. (2013). Genomic segments RNA1 and RNA2 of prunus necrotic ringspot virus codetermine viral pathogenicity to adapt to alternating natural Prunus hosts. *Mol. Plant Microbe Interact.* 26 515–527. 10.1094/MPMI-12-12-0282-R 23360459

[B8] Cui H. Wang A. (2016). Plum pox virus 6K1 protein is required for viral replication and targets the viral replication complex at the early stage of infection. *J. Virol.* 90 5119–5131. 10.1128/JVI.00024-16 26962227 PMC4859702

[B9] Cui H. Wang A. (2017). An efficient viral vector for functional genomic studies of Prunus fruit trees and its induced resistance to plum pox virus via silencing of a host factor gene. *Plant Biotechnol. J.* 15 344–356. 10.1111/pbi.12629 27565765 PMC5316922

[B10] Cui H. Wang A. (2019). The biological impact of the hypervariable N-terminal region of potyviral genomes. *Annu. Rev. Virol.* 6 255–274. 10.1146/annurev-virology-092818-015843 31299166

[B11] Cui X. Yaghmaiean H. Wu G. Wu X. Chen X. Thorn G. (2017). The C-terminal region of the turnip mosaic virus P3 protein is essential for viral infection via targeting P3 to the viral replication complex. *Virology* 510 147–155. 10.1016/j.virol.2017.07.016 28735115

[B12] Decroocq V. Salvador B. Sicard O. Glasa M. Cosson P. Svanella-Dumas L. (2009). The determinant of potyvirus ability to overcome the RTM resistance of *Arabidopsis thaliana* maps to the N-terminal region of the coat protein. *Mol. Plant Microbe Interact.* 22 1302–1311. 10.1094/MPMI-22-10-1302 19737103

[B13] Gallo A. Valli A. Calvo M. García J. A. (2018). A functional link between RNA replication and virion assembly in the potyvirus plum pox virus. *J. Virol.* 92:e02179-17. 10.1128/JVI.02179-17 29444942 PMC5899180

[B14] Gómez P. Sempere R. N. Amari K. Gómez-Aix C. Aranda M. A. (2010). Epidemics of tomato torrado virus, pepino mosaic virus and tomato chlorosis virus in tomato crops: do mixed infections contribute to torrado disease epidemiology? *Ann. Appl. Biol.* 156 401–410. 10.1111/j.1744-7348.2010.00397.x

[B15] Gómez P. Sempere R. N. Elena S. F. Aranda M. A. (2009). Mixed infections of pepino mosaic virus strains modulate the evolutionary dynamics of this emergent virus. *J. Virol.* 83 12378–12387. 10.1128/JVI.01486-09 19759144 PMC2786733

[B16] Harper S. J. Cowell S. J. Dawson W. O. (2017). Isolate fitness and tissue-tropism determine superinfection success. *Virology* 511 222–228. 10.1016/j.virol.2017.08.033 28888112

[B17] Ziebell H. MacDiarmid R. (2017). Prospects for engineering and improvement of cross-protective virus strains. *Curr. Opin. Virol.* 26 8–14. 10.1016/j.coviro.2017.06.010 28743041

[B18] Jacobson A. L. Duffy S. Sseruwagi P. (2018). Whitefly-transmitted viruses threatening cassava production in Africa. *Curr. Opin. Virol.* 33 167–176. 10.1016/j.coviro.2018.08.016 30243102

[B19] Maliogka V. I. Calvo M. Carbonell A. García J. A. Valli A. (2012a). Heterologous RNA-silencing suppressors from both plant- and animal-infecting viruses support plum pox virus infection. *J. Gen. Virol.* 93 1601–1611. 22513385 10.1099/vir.0.042168-0

[B20] Maliogka V. I. Salvador B. Carbonell A. Saenz P. León D. S. Oliveros J. C. (2012b). Virus variants with differences in the P1 protein coexist in a plum pox virus population and display particular host-dependent pathogenicity features. *Mol. Plant Pathol.* 13 877–886. 10.1111/j.1364-3703.2012.00796.x 22458641 PMC6638729

[B21] Mascia T. Gallitelli D. (2016). Synergies and antagonisms in virus interactions. *Plant Sci.* 252 176–192. 10.1016/j.plantsci.2016.07.015 27717453

[B22] Moreno A. B. López-Moya J. J. (2020). When viruses play team sports: mixed infections in plants. *Phytopathology* 110 29–48. 10.1094/PHYTO-07-19-0250-FI 31544593

[B23] Olspert A. Chung B. Y. W. Atkins J. F. Carr J. P. Firth A. E. (2015). Transcriptional slippage in the positive-sense RNA virus family Potyviridae. *EMBO Rep.* 16 995–1004. 10.15252/embr.201540509 26113364 PMC4552492

[B24] Pasin F. Simón-Mateo C. García J. A. (2014). The hypervariable amino-terminus of P1 protease modulates potyviral replication and host defense responses. *PLoS Pathog.* 10:e1003985. 10.1371/journal.ppat.1003985 24603811 PMC3946448

[B25] Pechinger K. Chooi K. M. MacDiarmid R. M. Harper S. J. Ziebell H. (2019). A new era for mild strain cross-protection. *Viruses* 11:670. 10.3390/v11070670 31340444 PMC6669575

[B26] Perez K. Yeam I. Kang B. C. Ripoll D. R. Kim J. Murphy J. F. (2012). Tobacco etch virus infectivity in *Capsicum* spp. is determined by a maximum of three amino acids in the viral virulence determinant VPg. *Mol. Plant Microbe Interact.* 25 1562–1573. 10.1094/MPMI-04-12-0091-R 23134519

[B27] Pruss G. Ge X. Shi X. M. Carrington J. C. Bowman Vance V. (1997). Plant viral synergism: the potyviral genome encodes a broad-range pathogenicity enhancer that transactivates replication of heterologous viruses. *Plant Cell* 9 859–868. 10.1105/tpc.9.6.859 9212462 PMC156963

[B28] Qin L. Shen W. Tang Z. Hu W. Shangguan L. Wang Y. (2021). A newly identified virus in the family Potyviridae encodes two leader cysteine proteases in tandem that evolved contrasting RNA silencing suppression functions. *J. Virol.* 95 e1414–e1420. 10.1128/JVI.01414-20 33055249 PMC7737751

[B29] Redondo E. Krause-Sakate R. Yang S. J. Lot H. Le Gall O. Candresse T. (2001). Lettuce mosaic virus pathogenicity determinants in susceptible and tolerant lettuce cultivars map to different regions of the viral genome. *Mol. Plant Microbe Interact.* 14 804–810. 10.1094/MPMI.2001.14.6.804 11386376

[B30] Revers F. García J. A. (2015). Molecular biology of potyviruses. *Adv. Virus Res.* 92 101–199. 10.1016/bs.aivir.2014.11.006 25701887

[B31] Rey C. Vanderschuren H. (2017). Cassava mosaic and brown streak diseases: current perspectives and beyond. *Annu. Rev. Virol.* 4 429–452. 10.1146/annurev-virology-101416-041913 28645239

[B32] Riechmann J. L. Cervera M. T. Garcia J. A. (1995). Processing of the plum pox virus polyprotein at the P3-6K1 junction is not required for virus viability. *J. Gen. Virol.* 76 951–956. 10.1099/0022-1317-76-4-951 9049341

[B33] Rodamilans B. Valli A. Mingot A. San León D. Baulcombe D. López-Moya J. J. (2015). RNA polymerase slippage as a mechanism for the production of frameshift gene products in plant viruses of the Potyviridae family. *J. Virol.* 89 6965–6967. 10.1128/JVI.00337-15 25878117 PMC4468506

[B34] Rodamilans B. Valli A. Mingot A. San León D. López-Moya J. J. García J. A. (2018). An atypical RNA silencing suppression strategy provides a snap-shot of the evolution of sweet potato-infecting potyviruses. *Sci. Rep.* 8:15937. 10.1038/s41598-018-34358-y 30374036 PMC6206096

[B35] Salvador B. Delgadillo M. O. Sáenz P. García J. A. Simón-Mateo C. (2008). Identification of plum pox virus pathogenicity determinants in herbaceous and woody hosts. *Mol. Plant Microbe Interact.* 21 20–29. 10.1094/MPMI-21-1-0020 18052879

[B36] Shen W. Shi Y. Dai Z. Wang A. (2020). The RNA-dependent RNA polymerase NIb of potyviruses plays multifunctional, contrasting roles during viral infection. *Viruses* 12:77. 10.3390/v12010077 31936267 PMC7019339

[B37] Sorel M. Garcia J. A. German-Retana S. (2014). The Potyviridae cylindrical inclusion helicase: a key multipartner and multifunctional protein. *Mol. Plant Microbe Interact.* 27 215–226. 10.1094/MPMI-11-13-0333-CR 24405034

[B38] Syller J. (2012). Facilitative and antagonistic interactions between plant viruses in mixed infections. *Mol. Plant Pathol.* 13 204–216. 10.1111/j.1364-3703.2011.00734.x 21726401 PMC6638836

[B39] Syller J. Grupa A. (2016). Antagonistic within-host interactions between plant viruses: molecular basis and impact on viral and host fitness. *Mol. Plant Pathol.* 17 769–782. 10.1111/mpp.12322 26416204 PMC6638324

[B40] Tatineni S. Elowsky C. Graybosch R. A. (2017). Wheat streak mosaic virus coat protein deletion mutants elicit more severe symptoms than wild-type virus in multiple cereal hosts. *Mol. Plant Microbe Interact.* 30 974–983. 10.1094/MPMI-07-17-0182-R 28840785

[B41] Tian Y. P. Valkonen J. P. (2013). Genetic determinants of potato virus Y required to overcome or trigger hypersensitive resistance to PVY strain group O controlled by the gene Ny in potato. *Mol. Plant Microbe Interact.* 26 297–305. 10.1094/MPMI-09-12-0219-R 23113714

[B42] Urcuqui-Inchima S. Haenni A. L. Bernardi F. (2001). Potyvirus proteins: a wealth of functions. *Virus Res.* 74 157–175. 10.1016/S0168-1702(01)00220-9 11226583

[B43] Wen R. H. Maroof M. S. Hajimorad M. R. (2011). Amino acid changes in P3, and not the overlapping pipo-encoded protein, determine virulence of soybean mosaic virus on functionally immune Rsv1-genotype soybean. *Mol. Plant Pathol.* 12 799–807. 10.1111/j.1364-3703.2011.00714.x 21726381 PMC6640218

[B44] White K. A. (2015). The polymerase slips and PIPO exists. *EMBO Rep.* 16 885–886. 10.15252/embr.201540871 26160653 PMC4552478

[B45] Wylie S. J. Adams M. Chalam C. Kreuze J. López-Moya J. J. Ohshima K. (2017). ICTV virus taxonomy profile: potyviridae. *J. Gen. Virol.* 98 352–354. 10.1099/jgv.0.000740 28366187 PMC5797945

[B46] Xiang C. Han P. Lutziger I. Wang K. Oliver D. J. (1999). A mini binary vector series for plant transformation. *Plant Mol. Biol.* 40 711–717. 10.1023/A:1006201910593 10480394

[B47] Yang K. Ran M. Li Z. Hu M. Zheng L. Liu W. (2018). Analysis of the complete genomic sequence of a novel virus, areca palm necrotic spindle-spot virus, reveals the existence of a new genus in the family Potyviridae. *Arch. Virol.* 163 3471–3475. 10.1007/s00705-018-3980-x 30136252

[B48] Yang K. Shen W. Li Y. Li Z. Miao W. Wang A. (2019). Areca palm necrotic ringspot virus, classified within a recently proposed genus Arepavirus of the family Potyviridae, is associated with necrotic ringspot disease in areca palm. *Phytopathology* 109 887–894. 10.1094/PHYTO-06-18-0200-R 30133353

[B49] Yang X. Li Y. Wang A. (2021). Research advances in potyviruses: from the laboratory bench to the field. *Annu. Rev. Phytopathol.* 59 1–29. 10.1146/annurev-phyto-020620-114550 33891829

[B50] Ziebell H. Carr J. P. (2010). Cross-protection: a century of mystery. *Adv. Virus Res.* 76 211–264. 10.1016/S0065-3527(10)76006-1 20965075

