# Supplementary material for: Biological and Molecular Characterization of Two Closely Related Arepaviruses and Their Antagonistic Interaction in Nicotiana benthamiana
Source: Front Microbiol. 2021 Oct 18;12:755156. doi: 10.3389/fmicb.2021.755156 (PMC8558625; doi:10.3389/fmicb.2021.755156)
Supplement: Supplementary Figure 1 — The construction strategy of five ANRSV-derived virus clones. [file Data_Sheet_1.PDF]

Supplementary Material

Supplementary Table 1 Primers list in this study

Primer Name	Sequence (5′→3′)	Use
RSV-V-F	ATATA***GGATCC***AAA***GAATTC***AAA***GTCGAC***GATCGTTCAAACATTTGGCA	
RSV-V-R	ATATA***ACGCGT***AAA***GCTAGC***AAA***AGGCCT***CTCCAAATGAAATGAACT	
1-F	AAATTAAAACAAATCAAAACAAACAAGAAAAGAAAAGA	
2-R	ATATA***ACGCGT***GAGCTGCTAAATTGAAGACT	
3-F	ATATA***ACGCGT***GGGGATTTTGTTCAGTTTTACAACT	
3-R	ATATA***GGATCC***TCCATGCATCCTGTCGCT	pRS
4-F	ATATA***GGATCC***ATTTAACTATGGGAAGACTGT	
4-R	ATATA***GAATTC***CCTGTACATATGCCACAGTGCT	
5-F	ATATA***GAATTC***GTTTACACACCAATTTACTGTGTGGAT	
5-R	ATATA***GTCGAC***TTTTTTTTTTTTTTTTTTTTACCTAAGGGTAGTAGGTTCATTGA	
SOE-GFP-2R	TCTTCTCCTTTACTCATGTCCATTTGAAATTCCTTACTTGCGTG	
SOE-GFP-3F	CAAGTAAGGAATTTCAAATGGACATGAGTAAAGGAGAAGAACT	pRS-G
SOE-GFP-4R	CCAATATCCATCTGGAACTCTTTGCTCGCTTTGTATAGTTCATCCATGC	
SOE-GFP-5F	AACTATACAAAGCGAGCAAAGAGTTCCAGATGGATATTGGGGAAGAGAG	
SOE-mCh-2R	TTGCTCACCATGTCCATTTGAAATTCCTTACTT	
SOE-mCh-3F	AATTTCAAATGGACATGGTGAGCAAGGGCGAGGAG	pRS-mCh
SOE-mCh-4R	CTCTTTGCTCGCCTTGTACAGCTCGTCCATGC	
SOE-mCh-5F	CGAGCTGTACAAGGCGAGCAAAGAGTTCCAGAT	
PCB301-F	TACCCGCCAATATATCCTGTC	
N-1F	GGTTTCAATTTAGAGTTGGAGCATCAATGAGCTCTGAAATC	pRS-G-N^{SS}
N-1R	ATTTCAGAGCTCATTGATGCTCCAACTCTAAATTGAAACCAAC	
2-F	ATATAGCTAGCCCACACTATTTACAAACG	
M1-1F	GGTTTCAATTTAAAGTTGGAGGAACATTGAGTGACGAAATC	
M1-1R	ATTTCGTCACTCAATGTTCCTCCAACTTTAAATTGAAACCAAC	pRS-G-M1^{SS}
M1-2F	CGGGTGGTAAAGAATTTCAAGCTGACTTCCTTGATGTCGAAC	
M1-2R	TCGACATCAAGGAAGTCAGCTTGAAATTCTTTACCACCCG	
M2-1F	GGAGGTAAAGAGTTTCAAGCTGATTTCCTCGATGTTGAAC	
M2-1R	GTTCAACATCGAGGAAATCAGCTTGAAACTCTTTACCTCCACC	
M2-2F	AGAAATGTCTTGAGTTCCAATCAGAACAAGTTTTAACAAAAGAG	pRS-G-M2^{SS}
M2-2R	TTTGTTAAAACTTGTTCTGATTGGAACTCAAGACATTTCTTG	
M3-1F	GAAATGTCTTGAGTTCCAAAGCAATCAAACACTCACAAAGG	
M3-1R	CCTTTGTGAGTGTTTGATTGCTTTGGAACTCAAGACATTTCTT	
M3-2F	CAGATTTCGCAAATTTTCAGATTAGGCGAGTTACACACGTAC	pRS-G-M3^{SS}
M3-2R	ACGTGTGTAACTCGCCTAATCTGAAAATTTGCGAAATCTGC	
C-1F	GATTTTGAAAACTTTCAGATGAGAAAAGTTGCCTTCTTAC	
C-1R	GTAAGAAGGCAACTTTTCTCATCTGAAAGTTTTCAAAATCTTCATTC	pRS-G-C^{SS}
SSV-C-R	ATATA**GTCGAC**TTTTTTTTTTTTTTTTTTTTACCTAAGGGTACTAGGTTCAGTGAG	
SS9000F*	AAGAGGGGAATACGTCAAAAAG	
SS9300R*	AGGGCCATGTTCATACTCA	
RS8900F*	ATTCCAACAGGGCCAAATAC	RT-PCR
RS9300R*	GGACCATGTTCATACTCACTAACAT	
RS-9200F	GAGCAATGCTTACGGATGGC	
RS-9350R	GATCATAGCATGGCCAGTGC	
SS-8600F	GGAACTCCCGTTCTAGATGAGTC	
SS-8700R	GTCCGCTTCCTCCATATCTCTG	real-time qPCR
Actin-145F*	AAAGACCAGCTCATCCGTGGAGAA	
Actin-145R*	TGTGGTTTCATGAATGCCAGCAGC	
Spe-SS-1900R	ACCAACTCTGGTGGGTGTAAGCT	
SS900F*	GATATACTCCACGTTGCAGATC	specific detection of ANRSV and ANSSV via RT-PCR
Spe-RS-1000R	GTGCACCAAGCAGCCATGTTG	

Note: The primers indicated in asterisks are referred to previous publications (Yang *et al*., 2018; Yang *et al*., 2019; Cui & Wang, 2016)

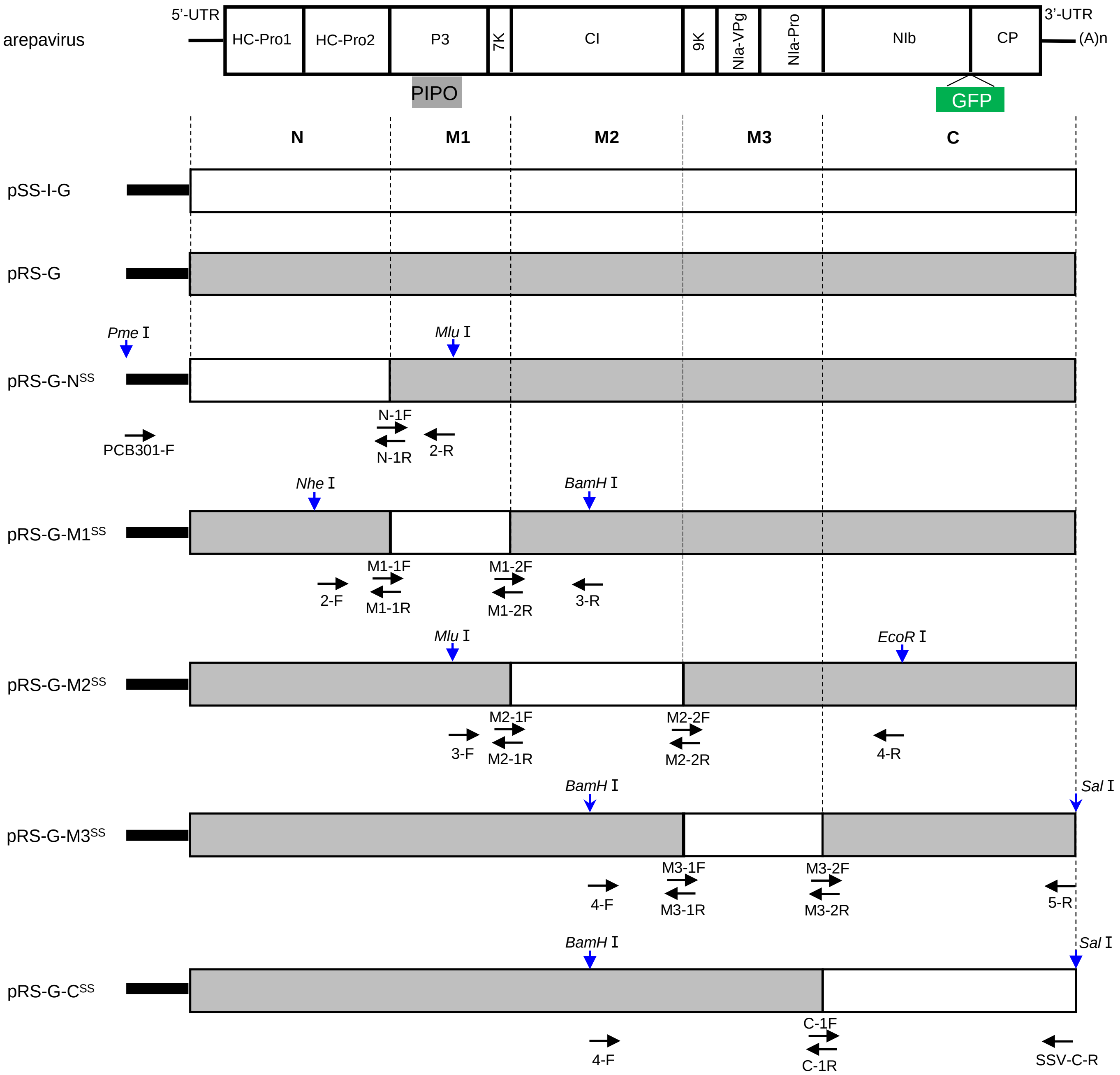

Supplementary Figure 1 The construction strategy of five ANRSV-derived virus clones. A total of five viral elements in ANRSV genome (i.e., 5'UTR-HCPro1-HCPro2, P3-6K1, CI, 9K-NIa, and NIb-CP) were individually replaced with their counterparts in ANSSV. Overlapping PCR assays and other conventional DNA manipulation techniques were employed produce these hybrid clones. For the generation of each hybrid clone, a pair of unique restriction endonuclease sites (indicated in blue arrows) were used to take out original element in pRS-G and insert back its counterpart in ANSSV. The counterpart fragments were obtained via overlapping PCR assay. The primers used for overlapping PCR are shown.

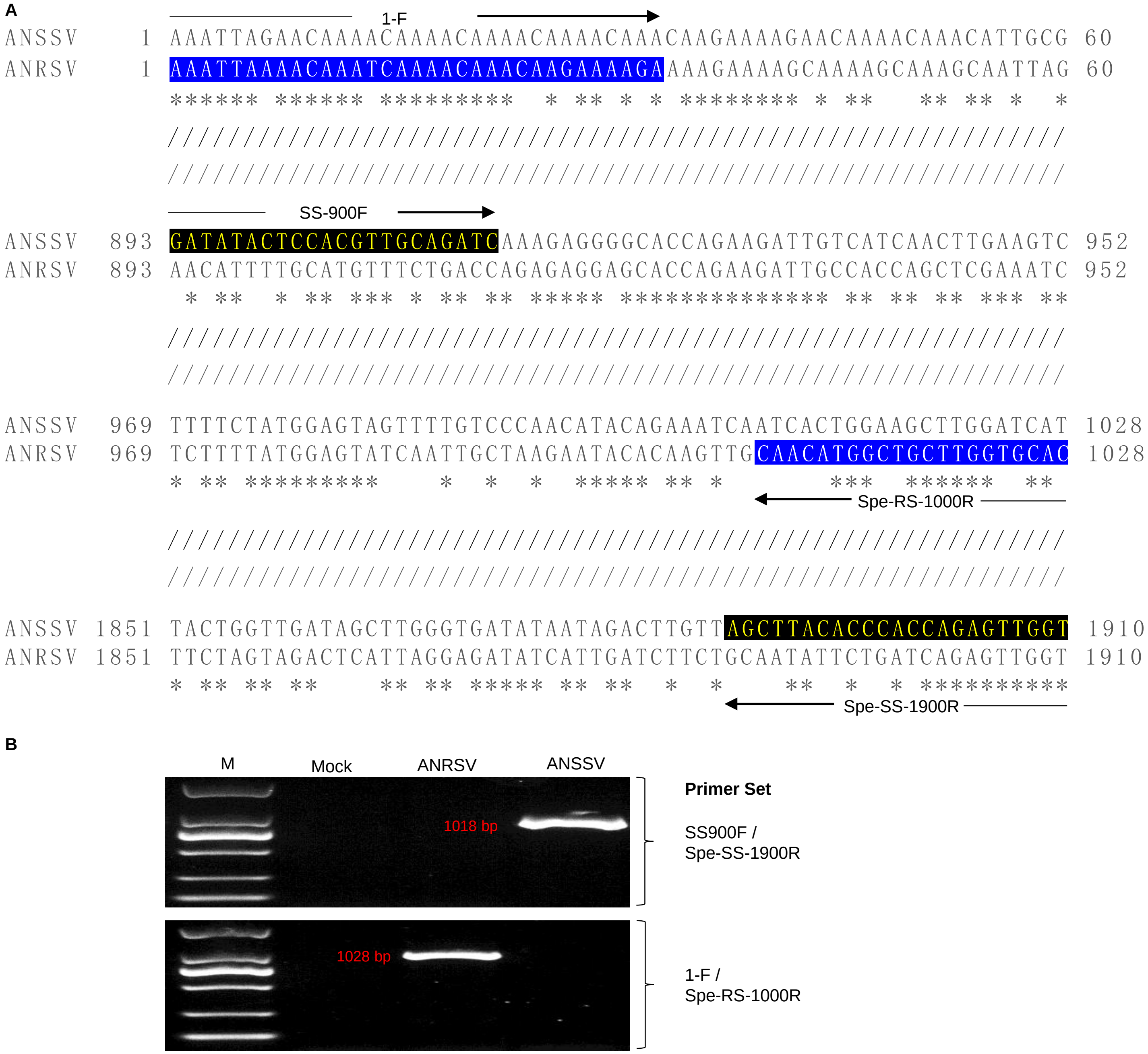

Supplementary Figure 2 Development of virus-specific primers for RT-PCR detection of ANSSV ANRSV. **(A)** The alignment of 5'UTR-HCPro1-HCPro2 sequences between ANSSV and ANRSV. Two specific primers Spe-RS-1000R (for ANRSV) and Spe-SS-1900R (for ANSSV) were designed. The Spe-RS-1000R and Spe-SS-1900R, respectively pairing with forward primers 1-F and SS-900R, are used for specific RT-PCR detection of ANRSV and ANSSV. **(B)** Specificity test of the above primer sets in RT-PCR detection of respective viruses. The fresh leaf tissues of individual *N. benthamiana* plant infected with ANRSV or ANSSV were sampled to evaluate the specificity of developed RT-PCR assays.

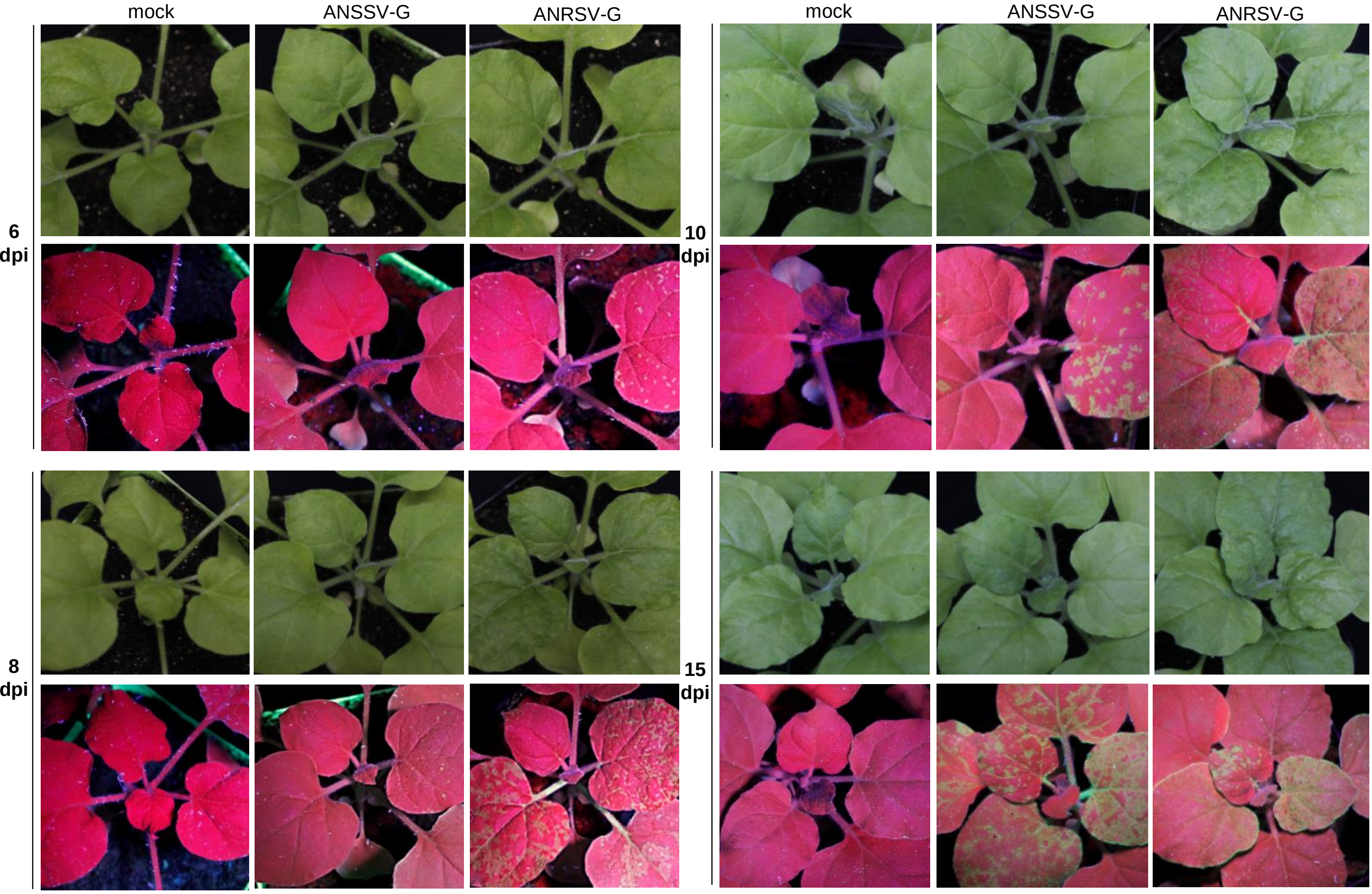

Supplementary Figure 3 Time-course observation on viral infection progression in *N. benthamiana* plants rub-inoculated with ANRSV-G and ANSSV-G. The representative photographs were taken at 6 dpri, 8 dpri, 10 dpri and 15 dpi.

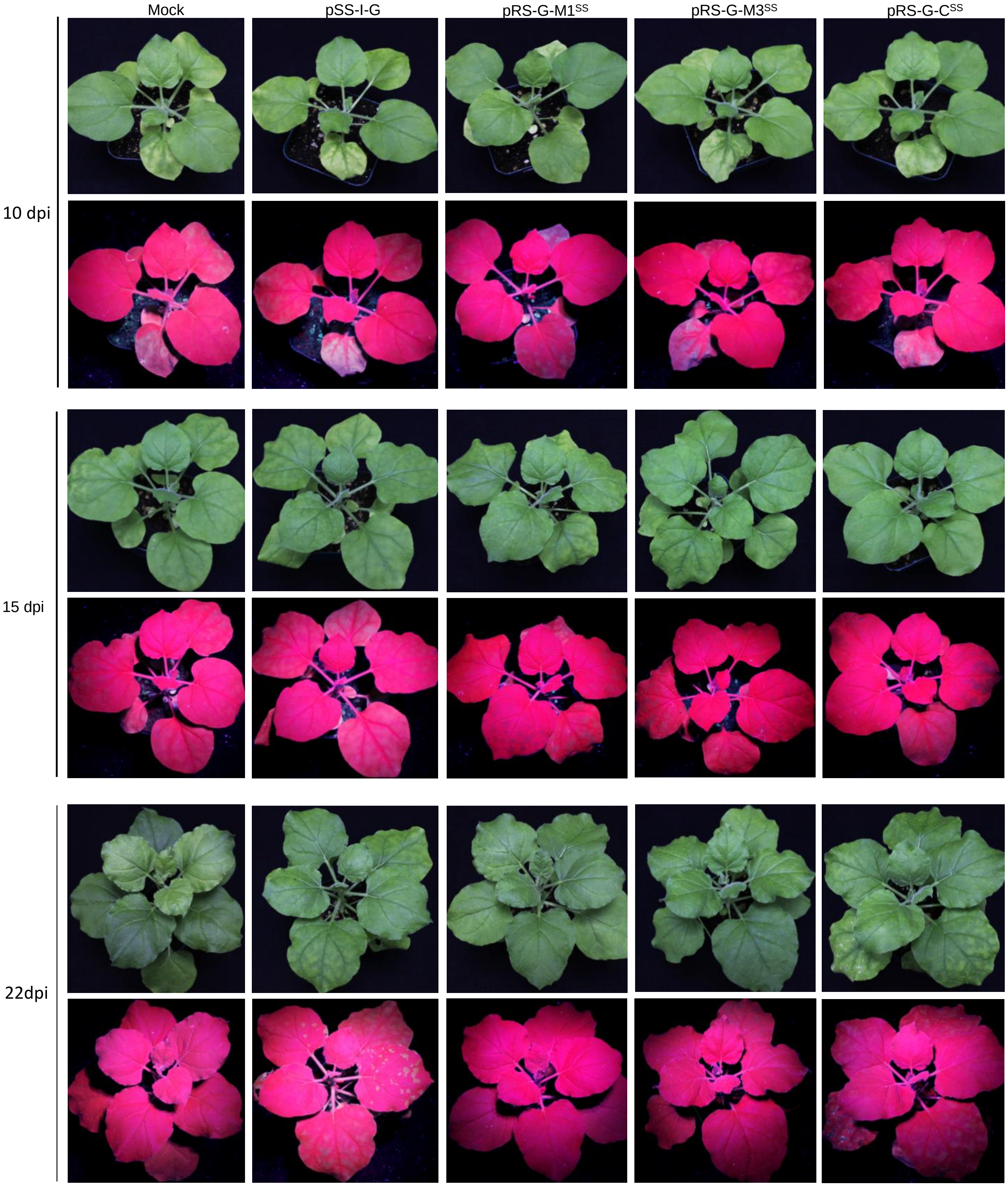

Supplementary Figure 4 Infectivity test of ANRSV-derived clones in *N. benthamiana*. The representative photographs of *N. benthamiana* plants agroinfiltrated with the indicated virus clones were taken at 10 dpi, 15 dpi and 22 dpi.
